# A vacuolar invertase gene *SlVI* modulates sugar metabolism and postharvest fruit quality and stress resistance in tomato

**DOI:** 10.1093/hr/uhae283

**Published:** 2024-10-02

**Authors:** Yu Wu, Haonan Chen, Mengbo Wu, Yuanyi Zhou, Chuying Yu, Qihong Yang, Filip Rolland, Bram Van de Poel, Mondher Bouzayen, Nan Hu, Yikui Wang, Mingchun Liu

**Affiliations:** Key Laboratory of Bio-Resource and Eco-Environment of Ministry of Education, College of Life Sciences, Sichuan University, Chengdu 610065, Sichuan, China; Key Laboratory of Bio-Resource and Eco-Environment of Ministry of Education, College of Life Sciences, Sichuan University, Chengdu 610065, Sichuan, China; Key Laboratory of Bio-Resource and Eco-Environment of Ministry of Education, College of Life Sciences, Sichuan University, Chengdu 610065, Sichuan, China; Key Laboratory of Bio-Resource and Eco-Environment of Ministry of Education, College of Life Sciences, Sichuan University, Chengdu 610065, Sichuan, China; Vegetable Research Institute, Guangxi Academy of Agricultural Science, Nanning 530007, Guangxi, China; Vegetable Research Institute, Guangxi Academy of Agricultural Science, Nanning 530007, Guangxi, China; Laboratory of Plant Metabolic Signaling, Department of Biology, KU Leuven, Kasteelpark Arenberg 31, 3001 Heverlee, Belgium; KU Leuven Plant Institute, KU Leuven, Kasteelpark Arenberg 31, 3001 Heverlee, Belgium; KU Leuven Plant Institute, KU Leuven, Kasteelpark Arenberg 31, 3001 Heverlee, Belgium; Molecular Plant Hormone Physiology Laboratory, Division of Crop Biotechnics, Department of Biosystems, KU Leuven, Willem de Croylaan 42, 3001 Heverlee, Belgium; Laboratoire de Recherche en Sciences Végétales—Génomique et Biotechnologie des Fruits—UMR5546, Université de Toulouse, CNRS, UPS, Toulouse-INP, 31320 Toulouse, France; College of Biology and Food Engineering, Anyang Institute of Technology, Anyang 455000, Henan, China; Vegetable Research Institute, Guangxi Academy of Agricultural Science, Nanning 530007, Guangxi, China; Key Laboratory of Bio-Resource and Eco-Environment of Ministry of Education, College of Life Sciences, Sichuan University, Chengdu 610065, Sichuan, China

## Abstract

Sugars act as signaling molecules to modulate various growth processes and enhance plant tolerance to various abiotic and biotic stresses. Moreover, sugars contribute to the postharvest flavor in fleshy fruit crops. To date, the regulation of sugar metabolism and its effect in plant growth, fruit ripening, postharvest quality, and stress resistance remains not fully understood. In this study, we investigated the role of tomato gene encoding a vacuolar invertase, hydrolyzing sucrose to glucose and fructose. *SlVI* is specifically expressed during the tomato fruit ripening process. We found that overexpression of *SlVI* resulted in increased leaf size and early flowering, while knockout of *SlVI* led to increased fruit sucrose content, enhanced fruit firmness, and elevated resistance of postharvest fruit to *Botrytis cinerea.* Moreover, the content of naringenin and total soluble solids was significantly increased in *SlVI* knockout fruit at postharvest stage. Transcriptome analysis showed a negative feedback regulation triggered by sucrose accumulation in *SlVI* knockout fruit resulting in a downregulation of *BAM3* and *AMY2,* which are critical for starch degradation. Moreover, genes associated with cell wall, cutin, wax, and flavonoid biosynthesis and pathogen resistance were upregulated in *SlVI* knockout fruit. Conversely, the expression levels of genes involved in cell wall degradation were decreased in knockout fruit. These results are consistent with the enhanced postharvest quality and resistance. Our findings not only provide new insights into the relationship between tomato fruit sucrose content and postharvest fruit quality, but also suggest new strategies to enhance fruit quality and extend postharvest shelf life.

## Introduction

Sucrose serves as a direct source of carbon and energy for plant metabolism and storage, but it can also act as a signaling molecule that interacts with different phytohormone signaling networks [1, 2]. Through these interactions, sucrose plays a pivotal role in regulating various cellular processes crucial for plant growth and development. These include, but are not limited to, embryo establishment, seed germination, lateral root induction, and meristem development, notably influencing cell division and expansion [3, 4]. Moreover, sugars play a central role in stress perception and signaling, modulating stress-induced gene expression and directing carbon allocation to adjust osmotic balance and scavenge reactive oxygen species. These integrated processes collectively aid in alleviating the detrimental effects of environmental stress on plant physiology [5–7].

Sugar metabolism in plants is a dynamic and complex process involving the loading and unloading and long-distance transport (source to sink) of sugars, and is largely controlled by sugar transporters and hydrolases [8]. The translocation of sugars from source tissues (e.g. leaves) to sink tissues (e.g. fruit) primarily involves passive mass flow within the phloem. However, active mechanisms are necessary for loading and unloading, requiring the transport of sugars across cell membranes. This transport is facilitated by various types of transporter proteins, including SUC/SUT sucrose-H^+^ symporters, proton-coupled MST (monosaccharide sugar transporter), and SWEET (Sugars Will Eventually be Exported Transporter) uniporters [9–11]. In sink tissue, sucrose is hydrolyzed into hexoses (glucose and fructose) or their nucleotide derivatives (UDP/ADP-glucose and fructose) through the enzymatic actions of invertases (INV) and sucrose synthases (SUS/SuSy), respectively, facilitating participation in diverse metabolic processes [12, 13]. Invertases can be classified into different isoforms, including acidic vacuolar invertases (VIN), cell wall invertases (CWIN), and neutral cytoplasmic invertases (CIN), based on their optimal pH, solubility, and subcellular localizations. Imported or symplastically unloaded sucrose can be hydrolyzed by CIN in the recipient cytoplasm or transported into vacuoles for subsequent hydrolysis into glucose and fructose by VIN [14]. Intracellular glucose and fructose can be allocated to various metabolic processes such as glycolysis and respiration, synthesis of polymers such as cellulose and starch, or storage in vacuoles, and/or act as signaling molecules, thereby modulating gene expression [15, 16]. The activities of VIN and CWIN are also modulated post-translationally by inhibitor proteins [17, 18].

The tomato fruit is an important sink organ with a very dynamic sugar metabolism. During the early stages of tomato fruit development, e.g. there is an important transient accumulation of starch that can reach levels of up to 20% of the fruit’s dry weight [19]. During fruit ripening, starch stored in the plastids is broken down into sugars, contributing to the soluble hexose level in the mature fruit [20, 21]. This transition, known as the starch–sugar interconversion, is facilitated by enzymes such as α-amylases and invertases [22, 23]. Glucose, fructose, and sucrose are the primary sugars found in ripe tomato fruit. Consequently, the accumulation of soluble sugars and their relative ratios play a significant role in shaping the flavor of postharvest tomatoes [24]. In recent years, studies employing RNA interference (RNAi) have provided valuable insights into the specific genes involved in sucrose metabolism and transport and their impact on various aspects of tomato plant physiology. Suppression of *SUS1* in tomato resulted in a noticeable reduced growth rate and reduced fruit set shortly after flowering. Besides, *SlSUS*-RNAi lines showed altered expression of genes associated with leaf morphology and auxin levels [25, 26]. Similarly, the targeted suppression of the cell wall invertase, *LIN5*, led to reduced glucose and fructose levels and detrimental effects on reproduction, including aberrant pollen morphology, decreased pollen viability and germination rate, and a decline in fruit set, size, and seed number [27]. This highlights the importance of sucrose hydrolysis in normal fruit development and fertility. In addition, functional studies on sugar transporters have also shown that inhibition of *SlSUT1* led to sucrose and starch accumulation in leaves due to blocked phloem loading, triggering premature leaf senescence while suppression of *SlSUT2* only affected tomato fruit and seed development with deficient pollen tube elongation, leading to the production of small, sterile fruit with a reduced seed number. Additionally, silencing of *SUT4* increased leaf sucrose export and increased drought tolerance of tomato plants [28, 29].

RNAi-mediated suppression of hexose transporters, such as *LeHT3*, led to a significant reduction in hexose (glucose and fructose) concentrations within the fruit, while photosynthetic rates and sucrose unloading from the phloem in leaves remained unaffected [30, 31]. Surprisingly, RNAi silencing of *SlSWEET7a* and *SlSWEET14*, which encode plasma membrane localized transporters, resulted in taller plants and larger fruit with enhanced sugar accumulation in mature fruit. This effect could be attributed to the observed increase in invertase activity and expression of other SWEET members. Overexpression of *SlSWEET11b* led to the redistribution of sugars in the stem, increases stem diameter, and accumulates more lignin. Further studies showed that the size, weight, and sugar content of fruit were significantly increased [32]. However, the specific role of the vacuolar invertases SlVI in regulating sucrose content and its impact on tomato plant physiology and postharvest quality of fruit are still not fully understood.

In this study, through integration of two transcriptome datasets, we revealed that the *SlVI* gene displays a distinct expression pattern during the tomato fruit ripening process. To further elucidate the role of SlVI in tomato plant growth and fruit quality, we conducted experiments involving overexpression of the *SlVI* gene and employed CRISPR/Cas9-mediated gene editing to generate *SlVI* knockout lines. Our analyses showed that overexpression of the *SlVI* not only resulted in increased chlorophyll content and leaf size, but also accelerated flowering and reproductive growth. Conversely, *SlVI* knockout lines produced fruit with higher soluble solids and naringenin chalcone content. Furthermore, *SlVI* knockout fruit exhibited higher firmness and extended shelf life. Gray mold infection demonstrated that *SlVI* knockout fruit displayed enhanced resistance to *Botrytis cinerea* compared to the wild type (WT). Moreover, the content of cellulose, hemicellulose, and protopectin (three main components of the cell wall) significantly increased in the *SlVI* knockout fruit. Further investigation revealed that the *SlVI* knockout pericarp exhibited a smaller and denser cell structure based on staining of paraffin transverse sections of tomato fruit. These findings collectively contribute to our understanding of the link between tomato fruit sucrose content and postharvest fruit quality.

## Results

### Tomato vacuolar invertase gene *SlVI* is specifically expressed during fruit ripening process

**Figure 1:**
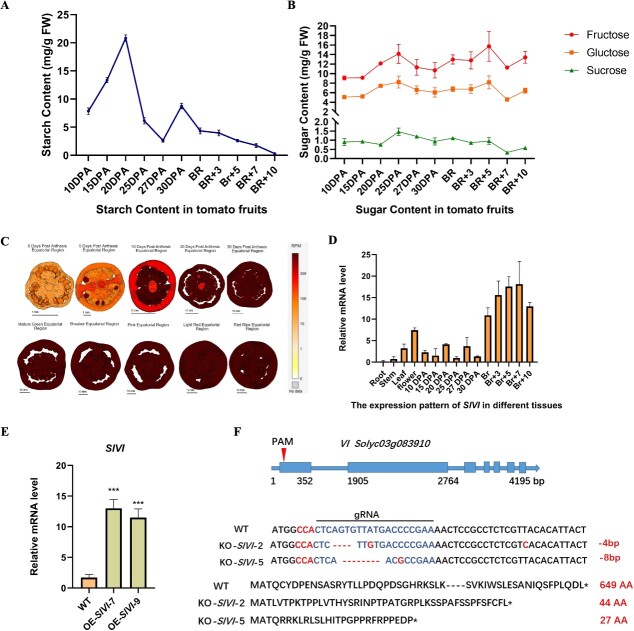
**Tomato vacuolar invertase (SlVI) is specifically expressed during fruit ripening. A,** Starch content at different fruit developmental and ripening stages of tomato fruit. **B,** Sugar content at different fruit developmental and ripening stages of tomato fruit. Under the same growth conditions, six fruits were collected in each stage, mixed, and ground as samples for determination, and each of them has three technical repetitions. **C,** Expression pattern images of *SlVI* gene from TEA *S. lycopersicum* M82 RNA-seq data. **D,** Relative *SlVI* transcript levels in different tissues and fruit development stages in Micro Tom WT tomato. DPA, days post-anthesis; Br, fruit at breaker stage. **E,** Relative SlVI transcript levels in WT and T1 generation SlVI overexpression lines in tomato leaves. Data are presented as means ± SD (*n* = 3). Asterisks indicate statistical significance using Student’s test ^*^P < 0.05, ^**^P < 0.01, and ^***^P < 0.001 and ns indicates no significant change when compared with WT. **F,** Schematic representation of CRISPR/Cas9-mediated gene editing at the first exon of the *SlVI* gene. Sanger sequencing showed two different base deletion mutations in T1 homozygous lines. The corresponding amino acid sequence indicated the production of a premature stop-codon and formation of truncated protein variants after editing. The asterisk represents the translation stop, the blue font indicates the sequence of the gRNA, and the red font the PAM, deletions, and mutations.

Starch serves as the principal storage carbohydrate in plants and plays a crucial role in modulating sugar homeostasis, particularly in response to fluctuating environments. Some fruits store starch early in fruit growth and undergo degradation during the ripening progress [33, 34]. To investigate the dynamics of starch and sugar content during fruit development and ripening, we utilized liquid chromatography and mass spectrometry (LC–MS) to measure these compounds at various developmental stages, ranging from 10 days post-anthesis (DPA) to 30 DPA, as well as different ripening stages: breaker (Br), Br + 3 (3 days postbreaker), Br + 5, Br + 7, and Br + 10 in Micro Tom tomato (Fig. 1a; Fig. S1). The results reveal two distinct phases of starch accumulation in tomato fruit. In the first phase (10–20 DPA), corresponding to fruit development and rapid growth, starch gradually accumulates, reaching its peak at 20 DPA, and then gradually degrades. During the second phase (27–30 DPA), another peak of starch accumulation is observed at 30 DPA, followed by degradation at the onset of ripening (Fig. 1a). Interestingly, the three main sugars also exhibit two accumulated peaks, occurring at 25 DPA and Br + 5 which coincide with the stages of starch degradation and consistently lag behind the peak of starch accumulation (Fig. 1b). These results suggest that tomato fruit initially stores carbon as starch during the early rapid growth stage and subsequently mobilizes it during later development and ripening stages. Furthermore, the simultaneous occurrence of starch degradation and sugar accumulation highlights the dynamic transformation of starch into sugars and other substances during tomato fruit development and ripening.

To further explore the metabolism and transport of sugar during tomato fruit ripening, we conducted an analysis of the expression of genes related to sucrose transport and metabolism in the transcriptome datasets of Micro Tom and revealed a specific increase in expression of a vacuolar invertase gene (*SlVI*, *Solyc03g083910*) during fruit ripening (Fig. 1c; Figs S2 and S3) [35]. Phylogenetic analysis showed the presence of just two vacuolar invertases, SlVIN1/VI and SlVIN2/LIN9 in tomato (Fig. S4). To validate the specific expression pattern of *SlVI* during fruit ripening, quantitative real-time polymerase chain reaction (RT-qPCR) analysis was performed in various tissues including root, stem, leaf, bud, flower, and fruit at different developmental and ripening stages. The results of the RT-qPCR analysis showed that *SlVI* displays high expression levels during fruit ripening stages (Fig. 1d). The combined evidence from the transcriptome data and tissue-specific expression analysis highlights the pronounced expression of *SlVI* during fruit ripening compared to other genes involved in sugar hydrolysis and transport. This observation suggests that SlVI may have an important role in regulating fruit sugar content.

### Overexpression of *SlVI* leads to earlier flowering and increased leaf size and chlorophyll content

To elucidate the function of SlVI in tomato sugar metabolism and investigate its impact on plant physiology and development, we generated both overexpression and knockout lines. Two independent overexpression lines driven by a CaMV35S promoter (OE-*SlVI*-7 and 9) show a significant increase in mRNA level of *SlVI* in leaves compared to the WT (Fig. 1e). Additionally, we obtained two Cas9-free homozygous knockout lines (KO-*SlVI*-2 and 5) by CRISPR/Cas9-mediated gene editing using a specific guide RNA targeting the first exon (Fig. 1f). Based on the Sanger sequencing results, it was shown that there were four and eight base pair deletions in the two knockout lines, respectively. These deletions resulted in premature stop codons and truncated protein versions (Fig. 1f).

**Figure 2:**
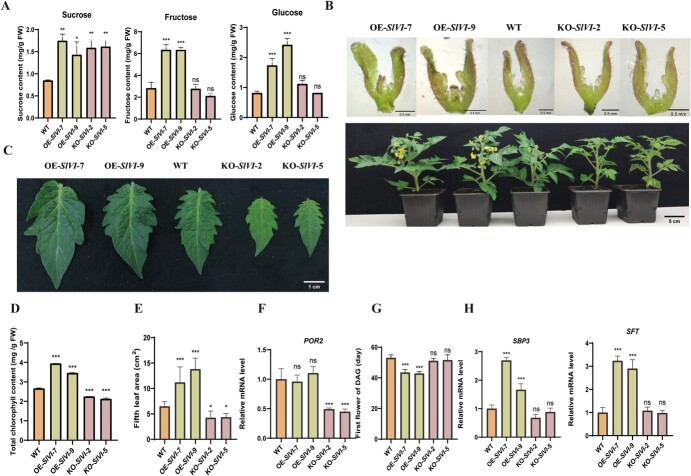
**Overexpression of *SlVI* leads to earlier flowering and increased leaf size and chlorophyll content. A,** Soluble sugar (sucrose, fructose, glucose) contents in WT, KO-*SlVI*, and OE-*SlVI* leaves. **B,** Microscopic photos of flower primordia in seedlings 10 days after germination (Bar = 0.5 mm) and the premature flowering phenotype of OE-*SlVI* plants (Bar = 5 cm). **C,** Phenotype of WT, KO-*SlVI*, and OE-*SlVI* lines fifth leaves 6 weeks after germination. Bar = 1 cm. **D,** Total chlorophyll content in WT, KO-*SlVI*, and OE-*SlVI* leaves. **E,** Areas of fifth leaf 6 weeks after germination. Leaves were photographed and areas measured by ImageJ. **F,** Relative *POR2* transcript levels in WT, KO-*SlVI*, and OE-*SlVI* leaves. **G,** Time from germination to first flower in WT, KO-*SlVI*, and OE-*SlVI* lines. **H,** Relative *SBP3* and *SFT* transcript levels in WT, KO-*SlVI*, and OE-*SlVI* leaves. Data are presented as means ± SD (A, *n* = 3; D, *n* = 3; E, *n* = 20; F, *n* = 3; G, *n* = 20; H, *n* = 3). Asterisks indicate statistical significance using Student’s test *P < 0.05, **P < 0.01, and ***P < 0.001 and ns indicates no significant change when compared with WT.

We first analyzed the effects of *SlVI* knockout and overexpression on the plant phenotype and sugar metabolism during vegetative growth. Surprisingly, both knockout or overexpression of this gene resulted in increased sucrose contents in tomato leaves. However, only the overexpression of *SlVI* significantly elevated the levels of sucrose hydrolysis products fructose and glucose (Fig. 2a). Interestingly, the overexpression lines exhibited darker green leaves and larger leaf size, while the knockout lines showed the opposite phenotype when compared with WT plants (Fig. 2c). Further analysis revealed that the chlorophyll content of the leaves of overexpression lines was significantly higher compared to WT. Conversely, the chlorophyll content in the knockout line was significantly lower than WT (Fig. 2d). Additionally, 6 weeks after germination, the fifth leaf of the tomato plant was selected for leaf size measurements.

The results showed that compared to the WT, the leaves of the knockout plants were indeed significantly smaller, whereas the leaves of the overexpression plants were larger (Fig. 2e). Consistently, expression of *POR2*, encoding protochlorophyllide oxidoreductase, a critical enzyme in chlorophyll synthesis, was significantly downregulated in knockout lines, while no significant change was observed in overexpression lines (Fig. 2f). In addition to the above-mentioned phenotypes, we also observed that the flowering time of overexpression plants was earlier than that of WT plants. Specifically, the expression level of *SBP3*(encoding an SPL/SBP-box protein), which is essential for the plant to initiate flowering and control flowering time [36], was significantly upregulated in overexpression lines (Fig. 2g, b, h). *SFT* induces early flowering in day-neutral plants and single-flower truss (*sft*) is a late-flowering mutant [37]. The expression level of *SFT* gene in overexpression plants was significantly higher than that in WT and knockout lines (Fig. 2h). These findings suggest that altering sugar metabolism, specifically through the overexpression of *SlVI*, may enhance chlorophyll content and leaf size and also shorten the transition from the vegetative to the reproductive phase in tomato.

### Knockout of *SlVI* significantly increases total soluble solids, sucrose, and naringenin content in postharvest fruit

**Figure 3:**
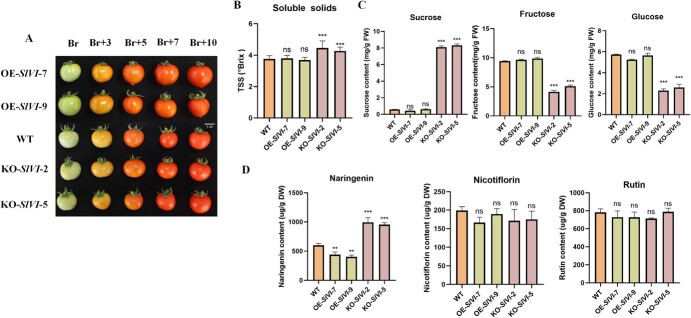
**Sucrose and naringenin levels are significantly increased in postharvest *SlVI* knockout fruit. A,** Phenotype of transgenic tomato fruit from breaker to 10 days after breaker stages. Bar = 1 cm. **B,** Content of soluble solids in WT, KO-*SlVI*, and OE-*SlVI* Br + 5 fruit. **C,** Soluble sugar (sucrose, fructose, glucose) contents in WT, KO-*SlVI*, and OE-*SlVI* Br + 5 fruit. **D,** Contents of three flavonoid compounds in WT, KO-*SlVI*, and OE-*SlVI* Br + 5 fruit. Data are presented as means ± SD (b, *n* = 20; (c–g, *n* = 3). Asterisks indicate statistical significance using Student’s test *P < 0.05, **P < 0.01, and ***P < 0.001 and ns indicates no significant change when compared with WT.

In addition to investigating the impact of *SlVI* expression on vegetative growth phenotypes, we also examined its effects on fruit development and ripening. Both pictures and quantification of the fruit ripening process duration (the time interval from anthesis to the breaker stage) and fruit firmness show that there is no significant difference between overexpression, knockout, and WT plants (Fig. 3a; Fig. S5a and b). To understand the effect of altered *SlVI* gene expression on fruit flavor and nutritional quality, we analyzed changes in total soluble solids, sugar, organic acid, and flavonoid content in transgenic fruit. The total soluble solids content in *SlVI* knockout fruit was higher than in WT, while no significant effect observed in fruit of overexpression lines (Fig. 3b). For the three main soluble sugars (sucrose, fructose, and glucose), we observed no significant change in fruit of the overexpression lines. At the same time, overexpression of *SlVI* did not affect fruit firmness, fruit shelf life, and *B. cinerea* resistance (S5b, c, d, e, f). However, in *SlVI* knockout fruit, sucrose levels were ~16 times higher than in WT. Consistent with the loss of invertase activity, the levels of glucose and fructose were decreased in *SlVI* knockout fruit (Fig. 3c). Organic acids are crucial factors that contribute to the flavor and taste of fruit since they provide acidity, balance sweetness, and enhance overall flavor perception. Investigation of the main organic acids, such as citric acid and malic acid, which contribute to the unique taste profile of tomato fruit, showed no significant differences among overexpression, knockout, and WT fruit (Fig. S6). Finally, flavonoids are essential secondary metabolites in fruit and contribute to various aspects of fruit quality and potential health benefits. We measured the content of three major flavonoids and found that the knockout fruit had a nearly 2-fold increase in naringenin levels compared to the WT, but no significant difference in the levels of nicotiflorin and rutin (Fig. 3d). These data indicate that the disruption of the *SlVI* gene increases the total soluble solids and flavonoids in tomato fruit and thus contributing to improving fruit taste and nutritional value. These findings shed light on the intricate relationships between sugar metabolism, fruit taste, and metabolite profiles in tomato fruit.

### Knockout of *SlVI* enhances fruit firmness by increasing the content of cell wall components

**Figure 4:**
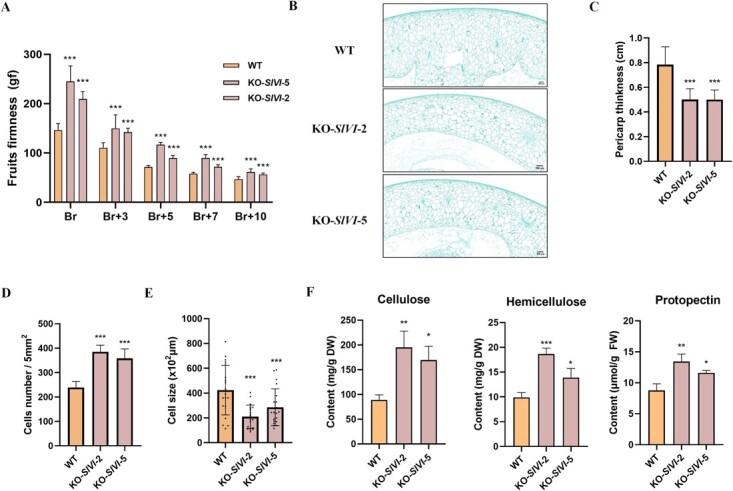
**Knocking out *SlVI* improves fruit firmness and cell wall components. A,** Fruit firmness in WT and KO-*SlVI* plants at different ripening stages. **B,** Safranin O-Fast Green staining of transverse paraffin-embedded tomato fruit sections at the Br stage in WT and KO-*SlVI*. Bars = 200 μm. **C,** Pericarp thickness at the Br stage in WT and KO-*SlVI*. Thickness of the pericarp in microscope images was measured using ImageJ. **D,** Cell number per 5 mm^2^ in WT and KO-*SlVI* pericarps was measured using ImageJ. **E,** Cell size in WT and KO-*SlVI* pericarps was measured using ImageJ. **F,** Contents of the three main cell wall components in WT and KO-*SlVI* fruit at the Br stage. Data are presented as means ± SD (A, E *n* = 20. C, D, F *n* = 3). Asterisks indicate statistical significance using Student’s test *P < 0.05, **P < 0.01, and ***P < 0.001 and ns indicates no significant change when compared with WT.

In postharvest storage, fruit firmness is a critical parameter that directly impacts the quality and shelf life of tomatoes. Maintaining optimal firmness levels helps to prevent mechanical damage, bruising, and decay during transportation, handling, and storage processes. Using a texture analyzer, we measured fruit firmness at different ripening stages. The results showed that the firmness was significantly increased in *SlVI* knockout lines compared to WT throughout the ripening stages ranging from breaker Br to Br + 10 (Fig. 4a). To further understand the reason for this increase in fruit firmness, we performed histological analysis on WT and *SlVI* knockout fruit. The microscopic images of the fruit’s equatorial cross-sections revealed that *SlVI* knockout fruit had a thinner pericarp compared to WT (Fig. 4b and c). However, upon counting the number of cells per 5 mm^2^ of the pericarp, we found that the knockout fruit had a significantly higher cell density and smaller cell size compared to the WT (Fig. 4d and e). These data indicated that although the pericarp thickness of the knockout lines was significantly reduced, the cells appeared less expanded and more densely organized. The increased cell density in the knockout fruit could potentially enhance cell wall strength and overall fruit firmness.

Cellulose, hemicellulose, and protopectin are the primary components of the cell wall in most plants. They provide structural support, protection, and rigidity to the cell wall [38]. To further investigate the impact of *SlVI* knocked out on cell wall composition, we quantified the content of cellulose, hemicellulose, and protopectin in the pericarp of WT and *SlVI* knockout fruit using a spectrophotometric method. In comparison to the WT, the pericarp of the knockout fruit displayed an ~2-fold increase in cellulose and hemicellulose content and a 1.5-fold increase in protopectin content (Fig. 4f). Increase of primary cell wall content may contribute to higher fruit firmness.

### Knockout of *SlVI* extends fruit shelf life and improves fruit *B. cinerea* resistance

**Figure 5:**
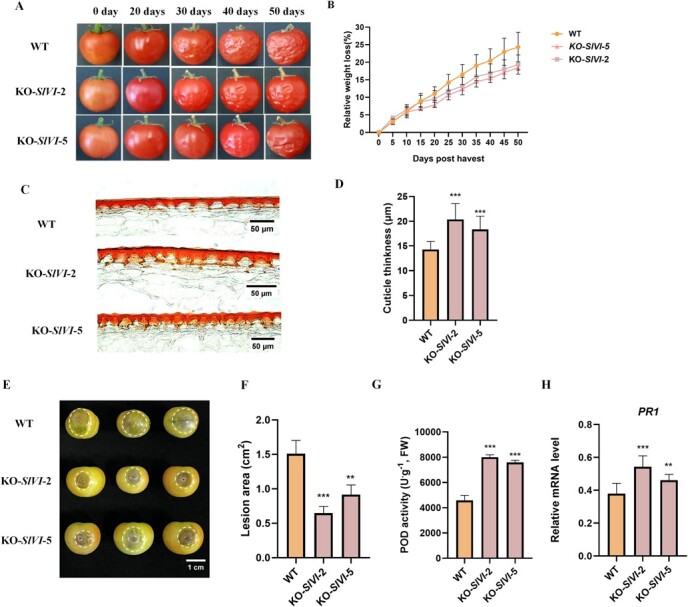
**Knockout of *SlVI* extends fruit shelf life and improves fruit *B. cinerea* resistance. A,** Phenotype of WT and KO-*SlVI* fruit during storage. **B,** Relative weight loss (weight/fresh weight) in WT and KO-*SlVI* fruit during storage. **C,** Micrographs of fruit epicarp transverse sections at Br stage in WT and KO-*SlVI* lines to visualize the cuticle. Bars = 50 μm. **D,** Fruit cuticle thickness in WT and KO-*SlVI* lines. Cuticle thickness was measured by ImageJ. **E,** Symptoms of WT and KO-*SlVI* fruit 48 h post-inoculation (hpi) with *B. cinerea.* White circles indicate the lesion margins. **F,** Lesion in WT and KO-*SlVI* fruit after infection. Lesion areas were measured by ImageJ. **G,** Antioxidant peroxidase activity in WT and KO-*SlVI* tomato fruit after inoculation with *B. cinerea.*  **H,** Relative expression levels of the disease resistance-related *PR1* gene in WT and KO-*SlVI* fruit after infection.

We performed a shelf life analysis to assess the postharvest quality of tomato fruit by measuring water loss (dehydration). Fruit in Br + 5 stage were selected, and their weight was recorded at regular intervals during a storage period of 50 days at room temperature. Interestingly, we found that *SlVI* knockout fruit exhibited less water loss symptoms, characterized by visible wrinkling and collapsing, indicating an improvement in shelf life compared with WT fruit (Fig. 5a). The weight loss data also revealed that WT fruit exhibited a higher rate of weight loss starting from 30 days postharvest, indicating extended shelf life of *SlVI* knockout fruit compared to WT (Fig. 5b). To further illustrate the water loss phenotype, we stained sections of fruit to visualize the cuticle thickness. As shown in Fig. 5c and d, *SlVI* knockout fruit have thicker cuticles compared to WT. Taken together, our findings suggest that knockout of *SlVI* positively influences fruit firmness by increasing the pericarp cell density and cell wall polymer content. In addition, knockout significantly extended the shelf life of the fruit likely by increasing the thickness of the cuticle.

Improving the postharvest storability of fruits and resistance to both biotic and abiotic stress is an important strategy to reduce food waste and economic losses in the fresh fruit industry. To evaluate the resistance of *SlVI* knockout fruit to pathogen infections, we conducted a *B. cinerea* inoculation experiment. At the Br stage, the fruit surfaces were inoculated with *B. cinerea*. After 48 h, we observed that the WT fruit produced larger lesion areas, indicating increased resistance to *B. cinerea* in the *SlVI* knockout fruit (Fig. 5e and f). Furthermore, we analyzed peroxidase (POD) activity and the expression of *SlPR1*, an established pathogen response gene, in fruit after *B. cinerea* infection. The results showed an increase in both POD activity and expression of *SlPR1* in *SlVI* knockout fruit, while these remained relatively low in WT (Fig. 5g and h). These findings suggest that knocking out *SlVI* gene in tomato enhances fruit resistance against *B. cinerea* infection, possibly through the activation of defense-related genes. These findings demonstrate that the alteration of sugar metabolism by *SlVI* knockout can confer improved resistance to *B. cinerea*.

### Transcriptome analysis revealed changes of sugar and postharvest quality-related genes in *SlVI* knockout fruit

**Figure 6:**
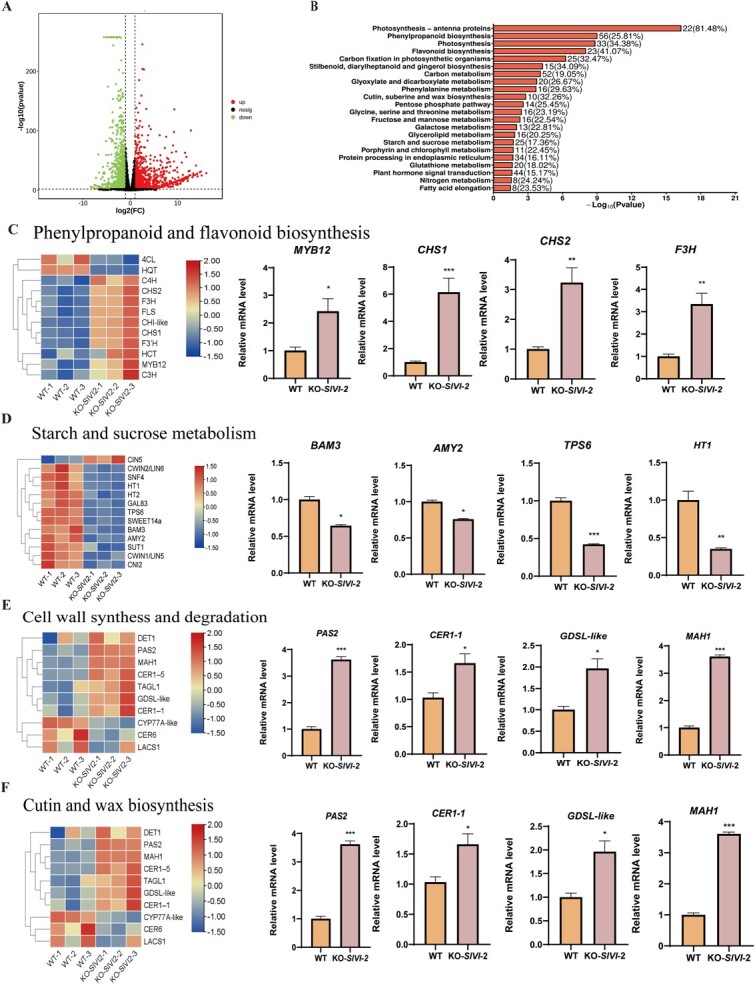
**RNA-seq profiling of WT and KO-*SlVI* fruit. A** Volcano plot displaying the differential genes expression profiles in WT and KO-*SlVI* samples. **B,** KEGG annotation of DEGs between WT and KO-*SlVI*-2. **C,** Heat map and RT-qPCR analysis of phenylpropanoid and flavonoid biosynthesis gene expression patterns. **D,** Heat map and RT-qPCR analysis of starch and sucrose metabolism-related gene expression patterns. **E,** Heat map and RT-qPCR analysis of cell wall synthesis and degradation-related gene expression patterns. **F,** Heat map and RT-qPCR analysis of cutin and wax biosynthesis-related gene expression patterns. Data are presented as means ± SD (*n* = 3). Asterisks indicate statistical significance using Student’s test *P < 0.05, **P < 0.01, and ***P < 0.001 and ns indicates no significant change when compared with WT.

To gain a deeper insight into how *SlVI* knockout affects tomato fruit sugar metabolism and fruit postharvest quality at molecular levels, we conducted global gene expression profiling using RNA sequencing (RNA-seq) analysis of *SlVI-KO* and WT fruit at Br + 5 stage. In total, we detected 3280 differently expressed genes (DEGs), of which 1655 were upregulated and 1625 were downregulated in fruit of *SlVI-KO* compared to WT. (Fig. 6a, Table S1). Kyoto Encyclopedia of Genes and Genomes (KEGG) annotation of these DEGs indicated that multiple metabolic pathways are significantly affected in *SlVI-KO* fruit. Genes involved in photosynthesis, phenylpropanoid and flavonoid biosynthesis, starch and sucrose metabolism, as well as cutin and wax biosynthesis were enriched in the DEGs (Fig. 6b). A gene ontology (GO) analysis also indicated that knockout of *SlVI* affected multiple biological process, including the carbohydrate metabolic process, response to oxidative stress, cell growth, and cell wall macromolecule catabolic process. In addition, some molecular functions, such as iron–ion binding, DNA-binding transcription factor activity, and transmembrane transporter activity are also affected (Fig. S7).

We first verified the impact on the expression of genes related to the metabolism of flavonoid, starch, and sucrose. As shown in the heat map, the trans-cinnamate 4-monooxygenase (*4CH*) gene, which is involved in the phenylpropanoid metabolic pathway, shows higher expression levels in fruit of *SlVI* knockout lines compared to WT (Fig. 6c). Transcription factor *SlMYB12*, which plays a key role in regulating the downstream flavonoid metabolic pathway, also exhibits higher expression level in the knockout fruit. Moreover, the higher expression levels of chalcone synthase genes (*CHS1* and *CHS2*) in the knockout fruit aligns with the increase in naringenin chalcone content. Furthermore, genes (*F3H* and *FLS*) related to the downstream steps in the flavonoid biosynthesis pathway are also highly expressed in the *SlVI* knockout tomato (Fig. 6c). This further supports the notion that disruption of the *SlVI* gene resulted in an increase in the production of flavonoid compounds (Fig. 3d). In addition, we also focused on the expression of genes related to starch degradation and sugar transport aimed to characterize the dynamic energy transport and transformation processes in fruit. The heat map analysis indicated a decrease in the expression of two genes, *BAM3* (Beta-amylase 3) and *AMY2* (Alpha-amylase 2), which play pivotal roles in the hydrolysis of starch into maltose and shorter oligosaccharides, respectively (Fig. 6d). This decline could potentially be attributed to the substantial accumulation of sucrose observed in *SlVI* knockout fruit, leading to negative feedback mechanism in energy conversion pathways. Furthermore, a significant decrease in the expression of *TPS6* (encoding a trehalose-6-P synthase-like protein) and *HT1* (encoding a hexose transporter) was observed in *SlVI*-KO fruit (Fig. 6d).

To investigate the changes in fruit texture and firmness at molecular levels, we examined the expression levels of cell wall synthesis and degradation related genes. The *SlVI* knockout fruit exhibited increased expression levels of xyloglucan endotransglucosylases/hydrolases (*XTHs*) and pectin methylesterases (*PMEs*) (Fig. 6e), suggesting an enhanced cell wall synthesis in *SlVI*-KO fruit. In contrast, the expression levels of poly-galacturonases (*PGs*) and cellulases (*CEL1*, and *CEL2*) involved in cell wall degradation were decreased in *SlVI*-KO fruit (Fig. 6e). This data is consistent with the extended shelf life and higher firmness observed in the knockout fruit (Figs 4a, 5a and b). In response to the remarkable reduction in water loss rate observed in knockout fruit during postharvest storage compared to WT, we checked the expression of genes associated with the fruit cuticle wax biosynthesis. Notably, the expression levels of *SlCER1-1*, a key gene involved in the biosynthesis of the precursor for cuticular wax components, were higher in *SlVI* knockout fruit compared to WT [39]. Additionally, *PAS2*, known to function in lipid metabolism or cuticle remodeling and, contributing to the formation and maintenance of the cuticular in tomato fruit, also exhibited increased expression in the knockout fruit [40] (Fig. 6f). In addition, the expression levels of the *GDSL-like* gene involved in tomato lipid metabolism and defense response and the cytochrome P450 enzyme *MAH1* involved in cuticle wax formation also significantly increased [41, 42] (Fig. 6f). These results indicate that sugar transport in fruit is a highly dynamic and balanced process, where changes in one gene can trigger a cascade of alterations in other genes involved in the different metabolic pathways.

### Transcriptional regulation of *SlVI* by ripening-related transcription factors

**Figure 7:**
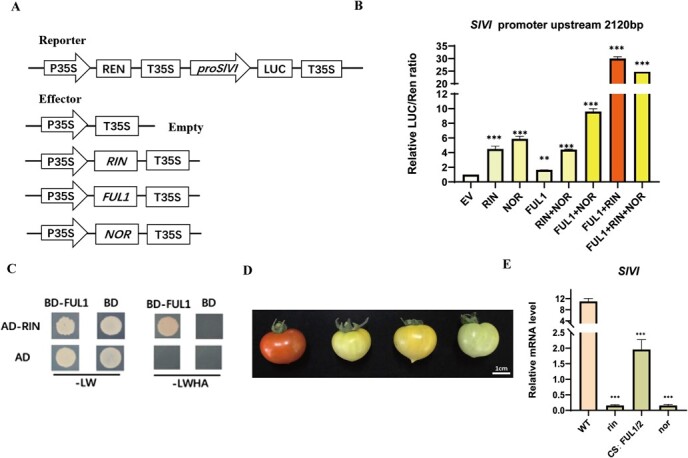
**Transcriptional regulation of *SlVI* by ripening-related transcription factors. A,** A schematic illustration of the LUC reporter and effector constructs used in transient expression assays. **B,** The activation of *SlVI* by ripening-related transcription factors. LUC, firefly luciferase; REN, Renilla luciferase. **C,** Analysis of the interaction between RIN and FUL1 in yeast system. **D** Phenotype of rin, nor mutant, and co-silence *FUL1/2* Br + 5 fruit. **E,** Relative expression level of *SlVI* genes in rin, nor mutant, and co-silence *FUL1/2* fruit. Data are presented as means ± SD (*n* = 3). Asterisks indicate statistical significance using Student’s test *P < 0.05, **P < 0.01, and ***P < 0.001 and ns indicates no significant change when compared with WT.

To investigate whether ripening-related transcription factors regulate the fruit-specific expression of the *SlVI* gene, we conducted a dual luciferase reporter assay. The results showed that the transcription factors RIN, FUL1, and NOR can bind to the promoter of the *SlVI* gene and activate its transcription, with activation levels approximately two to five times higher than the control. Furthermore, when RIN and FUL1 were co-expressed with the *SlVI* promoter, the activation effect increased to 30 times, suggesting a synergistic effect rather than a mere additive effect. However, we observed that RIN and NOR, as well as NOR and FUL1, do not exhibit synergistic effects (Fig. 7a and b). Yeast two-hybrid experiments further revealed that RIN and FUL1 can interact with each other, whereas NOR and FUL1 cannot (Fig. 7c). Due to strong auto-activation effects, we were unable to determine the interaction between RIN and NOR. To further support the conclusion that the specific expression of the *SlVI* gene in fruit is regulated by ripening-related transcription factors, we measured the expression of the *SlVI* gene in *rin*, *nor* mutants, and co-silenced *FUL1/2* fruit (Fig. 7d and e). The results showed that the expression level of the *SlVI* gene in these mutants was significantly lower compared to WT fruit.

## Discussion

Sucrose serves as a vital carbon and energy source for plant growth and development, participating in numerous biochemical reactions through a series of metabolic processes. Additionally, sugars such as sucrose, glucose, and fructose also act as signaling molecules that can more directly regulate various physiological and developmental processes in response to changing environmental conditions [15, 43]. Ripening-specific vacuolar invertase (SlVI) hydrolyzes sucrose into glucose and fructose during tomato fruit ripening. In our study, we observed a remarkable increase in sucrose and decrease in glucose and fructose content in *SlVI* knockout fruit without significant changes in the fruit ripening process (Fig. 3a–c; Fig. S5). For flavonoid metabolism pathways, *SlVI* knockout fruit exhibited a nearly 2-fold increase in naringenin content compared to the WT (Fig. 3d). From the transcriptome data, it is evident that many genes involved in the phenylpropanoid and flavonoid metabolic pathways are significantly upregulated in the knockout fruit (Fig. 6c). These findings align with previous studies demonstrating that sucrose promotes the accumulation of flavonoids in *Arabidopsis*, which may be mediated by DELLA protein stabilization [44, 45]. Plant cell wall metabolism is a multifaceted process, intricately regulated by various genes with distinct functions, even within the same gene family [38]. Texture analysis revealed a significant increase in fruit firmness in the knockout fruit and higher relative content of cell wall components in pericarp tissue compared to WT fruit (Fig. 4a and f). According to previous studies, overexpressing *SlXTH1* or knockout of *SlXTH5* in tomato can increase fruit firmness, while silencing of *SlPME2.1* in tomato fruit resulted in an increased rate of softening during ripening, which is consistent with our transcriptome data related to cell wall synthesis and degradation (Fig. 6e) [46, 47]. Moreover, simultaneous inhibition of *SlPG* and *SlEXP1*, or inhibition of *SlPL*, has been demonstrated to reduce cell wall disassembly and elevate levels of cellulose and hemicellulose. This impedes fruit softening and enhances resistance to *B. cinerea* [48, 49]. In our *SlVI* knockout fruit the substantial downregulation of these genes, as depicted in the heat map, may closely correlate with the increased content of cell wall components and the improved resistance to *B. cinerea* (Figs 4f, 5e). Besides, increased content of flavonoids in tomato fruit also has a positive effect on resistance to *B. cinerea* infection [50]. This suggests a synergistic interplay between flavonoids and cell wall composition in contributing to the enhanced resistance of *SlVI* knockout fruit against *B. cinerea*. Histological analysis demonstrated that the knockout fruit exhibited smaller but densely arranged cells (Fig. 4b, d, e). While there are relatively few reports directly linking endogenous sugar content to cell development and expansion, it is well established that sugar can exert regulatory effects on these processes by modulating plant hormone levels [51, 52]. From our transcriptome data and RT-qPCR validation, it can be concluded that key genes involved in multiple hormone signaling pathways are altered in knockout fruit (Fig. S8). However, the precise molecular mechanisms underlying these observations require further investigation.

Consistent with the previous findings that hexokinase, which is involved in the regulation of flowering-related genes through miR156, may control the transition from juvenile to adult stages in *Arabidopsis* [52], *SlVI* overexpression lines were characterized by increased hexose content in leaves and showed an early-flowering phenotype. However, the relationship between hexokinase activity and miR156 expression in *Arabidopsis* and tomato remains to be validated in the future. Additionally, we investigated the expression levels of *SlSBP3* and *SlSFT*, known positive regulators of flowering [36]. As expected, the expression levels of these two genes also increased in the *SlVI*-overexpressing lines (Fig. 2b, g, h). These findings suggest that altering sugar metabolism can accelerate the vegetative growth stage and promote early flowering, possibly through altered hexokinase levels and signaling.

In conclusion, our study highlights the importance of sucrose metabolism in tomato fruit development, flavor, texture, and postharvest quality. Disruption of the *SlVI* gene increased sucrose and naringenin content and improved fruit firmness by increasing endocarp cell wall polymer content, and coincided with enhanced resistance to *B. cinerea* infection. These findings expand our understanding of the molecular mechanisms underlying fruit development and ripening processes regulated by sucrose metabolism. Further research is warranted to unravel the intricate regulatory networks and molecular interactions involved in sugar and energy sensing and signaling and the modulation of fruit quality traits. The findings of this study provide valuable insights for the development of strategies aimed at improving fruit quality and postharvest storage of tomatoes.

## Materials and methods

### Plant material and growth conditions

Tomato plants (*Solanum lycopersicum* cv Micro Tom) were cultivated in a controlled greenhouse environment following standard culture conditions descried in Deng et al. [53].

### Plasmid construction and plant transformation

The full coding sequence (CDS) of the *SlVI* gene (*Solyc03g083910*) was integrated into the binary plant expression vector pBI121 and driven by constitutive CaMV35S promoter with a kanamycin resistance gene for selection of transgenic plants. To facilitate genome editing and generate knockout, the CRISPR/Cas9 vector, named BGFastCas9-plant, was obtained from BioGround Corp company and modified accordingly. The CRISPR-P 2.0 online tool (http://cbi.hzau.edu.cn/crispr/) was utilized to design a specific target site for the *SlVI* gene. Primers required for the construction of the CRISPR/Cas9 system are listed in Table S1. The constructed *SlVI* overexpression and CRISPR/Cas9 vectors were introduced into *Agrobacterium tumefaciens* strain GV3101 and using Agrobacterium-mediated methods.

### RNA extraction and RT-qPCR analysis

Total RNA was extracted from various tomato tissues using a plant RNA extraction kit (BIOFIT, Chengdu, China). First-strand cDNA was reverse-transcribed from 1 μg of total RNA using the PrimeScriptTM RT reagent kit (AK4201; Takara Bio, Kusatsu, Japan) according to the manufacturer’s instructions. For RT-qPCR, the BG0014 SYBR Prime qPCR Set (Fast HS; BioGround Corp company, Chongqing, China) was employed. The RT-qPCR experiments were performed on a Bio-Rad CFX96 Real-Time PCR System. The RT-PCR program and data analysis procedures followed the established method as described in previous studies [54]. The primer sequences used for RT-qPCR in this study are provided in Supplemental Table S1 and using Actin (*Solyc11g005330*) as the reference gene. All tissues were obtained from tomato plants grown under the same growth conditions, and each sample included three technical replicates.

### Chemical compound content analysis

To extract starch from tomato fruit powder, a starch detection kit from Solarbio, Beijing, China, was used following the manufacturer’s provided instructions. For the preparation of sugar standard solutions, fructose, glucose, and sucrose were individually dissolved in a mixture of 50% acetonitrile (ACN)/H2O at various concentrations ranging from 0.5 to 1 mg/ml. Similarly, citric acid and malic acid standards were dissolved in a 50% methanol/H2O solution at the same concentrations. Tomato fruit powder was first dissolved in the same solution with standards at a ratio of 0.1 g/ml and diluted 100 times and filtered using a 0.45-μm PVDF syringe filter for analysis. All analyses were performed using the SCIEX Triple Quad 5500 LC–MS/MS System. For sugar and organic acid analysis, ACQUITY UPLC BEH Amide Columns (1.7 μm, 2.1 × 100 mm, made in Ireland) were used to separate and quantify the compounds. Flavonoid metabolites were quantified following the method described by [54]. All standards used in these analyses were purchased from Shanghai Yuanye Bio-Technology (Shanghai, China).

### Fruit firmness and shelf life tests

To measure fruit firmness, a TA.XTC-18 texture analyzer (Bosin Tech, Shanghai, China) was utilized with a P/2 columnar probe of 2 mm diameter. More than 20 fruits at the Br + 5 stage were selected for the firmness measurements. The test parameters and calculation method were carried out as previously described in the study by You et al. [54]. For shelf life testing, fruit at the Br + 5 stage were harvested and placed in a plant climate chamber set at room temperature conditions (25°C, 55–60% relative humidity). The physiological loss of water (PLW) was calculated by measuring the weight loss per fruit every 5 days of storage. The appearance and quality of the fruit were visually assessed and photographed every 10 days throughout the storage period. At least 10 fruits were collected from each transgenic line for analysis and comparison.

### Cytological assessment and main cell wall components content determination

Tomato BR stage fruit was harvested for Safranin O-Fast Green staining (G1101, Wuhan Servicebio Technology Co., Ltd, China) and the method is consistent with the previous description [54]. For the three main cell wall components content, cellulase, hemicellulose, and protopectin content assay kits were purchased from Solarbio (Beijing Solarbio Science & Technology Co., Ltd) and using the spectrophotometer according to the manufacturer’s instructions.

### *Botrytis cinerea* resistance analysis

The pathogen *B. cinerea* was collected and grown on PDA medium at 20°C. Tomato fruit at BR stage was inoculated with mycelial plugs and stored at room temperature with relative high humidity for phenotype observation and further experimentation [55]. Infection symptoms were photographed and calculated at 48 h after inoculation using Image J.

### Transcriptome profiling

In our study, we performed RNA-seq to analyze the transcriptome profiles of tomato fruit at the BR + 5 stage for both the WT and KO-*SlVI*-2 lines. RNA-Seq liBrary construction and high-throughput sequencing were conducted by Biomarker Technologies (Beijing, China). The liBrary preparations were sequenced on an Illumina Novaseq 6000 platform, and 150-bp paired-end reads were generated. The liBrary quality was then assessed using an Agilent Bioanalyzer 2100 system and sequenced on an Illumina Hiseq X-Ten platform to obtain raw reads. DEGs were found using a significance threshold of a log 2-fold change of ±1. KOBAS software was used to test the statistical enrichment of DEGs in KEGG pathways. Pathways with the *P* < 0.05 were significantly enriched. GO analysis is a method used for gene annotations from a database Gene Ontology Consortium (http://geneontology.org/).

### Yeast two-hybrid assay

The full-length coding sequences of *RIN*, *NOR*, and *FUL1* were cloned into the pGADT7 and pGBKT7 vectors, respectively. These constructs were then transformed into the AH109 yeast strain. Yeast transformants were cultured on Synthetic dropout (SD) medium lacking Leu and Trp (SD-Leu-Trp) for 2–4 days to select for positive transformants. To confirm the interaction between the two proteins, selection medium plates (−Leu, −Trp, -His, −Ade) were utilized. Growth of yeast colonies on these plates indicated an interaction between the proteins.

### Dual-luciferase reporter assay

The promoter sequence of the *SlVI* (*Solyc03g083910*) gene was obtained from the tomato genome website (https://solgenomics.net/). The CDS sequences of ripening related transcription factors RIN, NOR, and FUL1 were cloned into a plant expression vector as effectors. Using transient transformation of tobacco leaves and test method were carried out as previously described in the study by You et al. [53].

## Supplementary Material

Web_Material_uhae283

## Data Availability

The authors confirm that all the experimental data are available and accessible via the main text and/or the supplemental data.
