# Correction to: A vacuolar invertase gene *SlVI* modulates sugar metabolism and postharvest fruit quality and stress resistance in tomato

**DOI:** 10.1093/hr/uhaf180

**Published:** 2025-07-28

This is a correction to: Yu Wu, Haonan Chen, Mengbo Wu, Yuanyi Zhou, Chuying Yu, Qihong Yang, Filip Rolland, Bram Van de Poel, Mondher Bouzayen, Nan Hu, Yikui Wang, Mingchun Liu, A vacuolar invertase gene *SlVI* modulates sugar metabolism and postharvest fruit quality and stress resistance in tomato, *Horticulture Research*, Volume 12, Issue 1, January 2025, 10.1093/hr/uhae283.

In the originally published version of the article, Figure 6E inadvertently contained an incorrect heatmap and RT-qPCR analysis of cell wall synthesis and degradation-related gene expression patterns. Figure 6 should read:

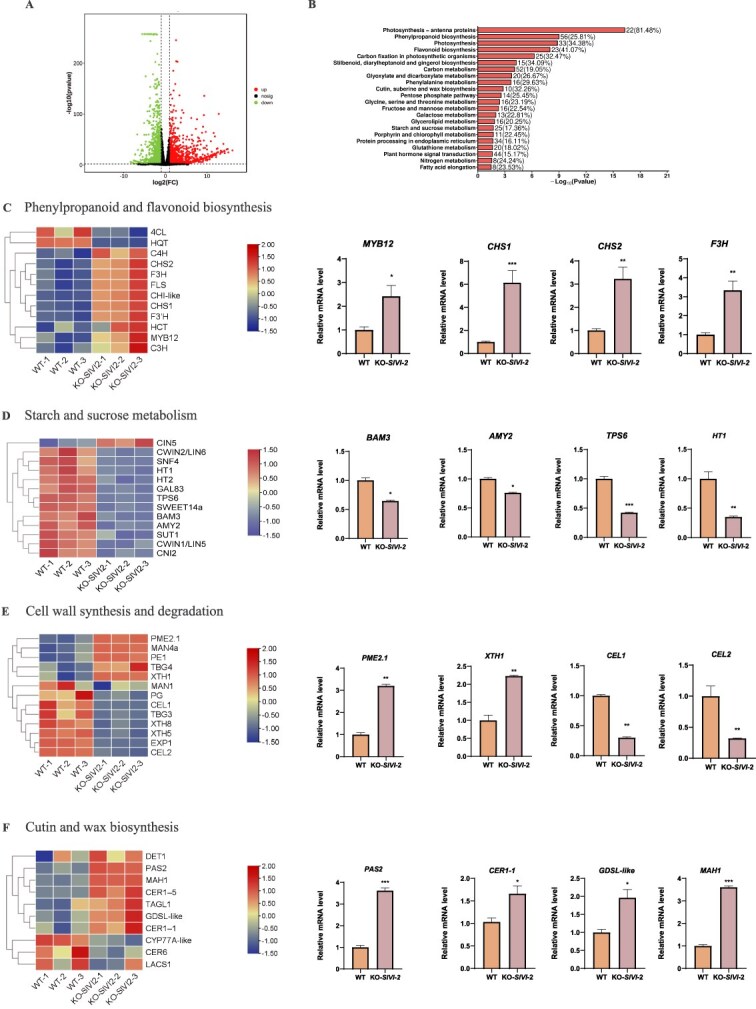



instead of:


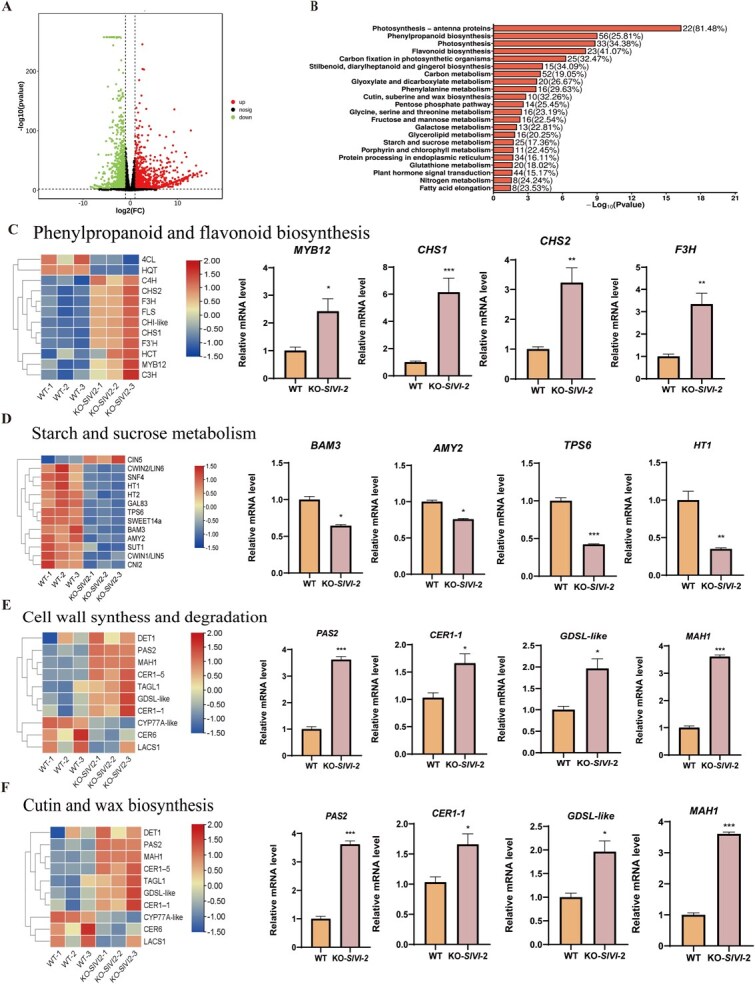


The revised figure reflects the experimental data.

