# Supplementary material for: A vacuolar invertase gene SlVI modulates sugar metabolism and postharvest fruit quality and stress resistance in tomato
Source: Hortic Res. 2024 Oct 2;12(1):uhae283. doi: 10.1093/hr/uhae283 (PMC11758369; doi:10.1093/hr/uhae283)
Supplement: Web_Material_uhae283 [file web_material_uhae283.zip › Supplment Figures.pptx]

## Slide 1
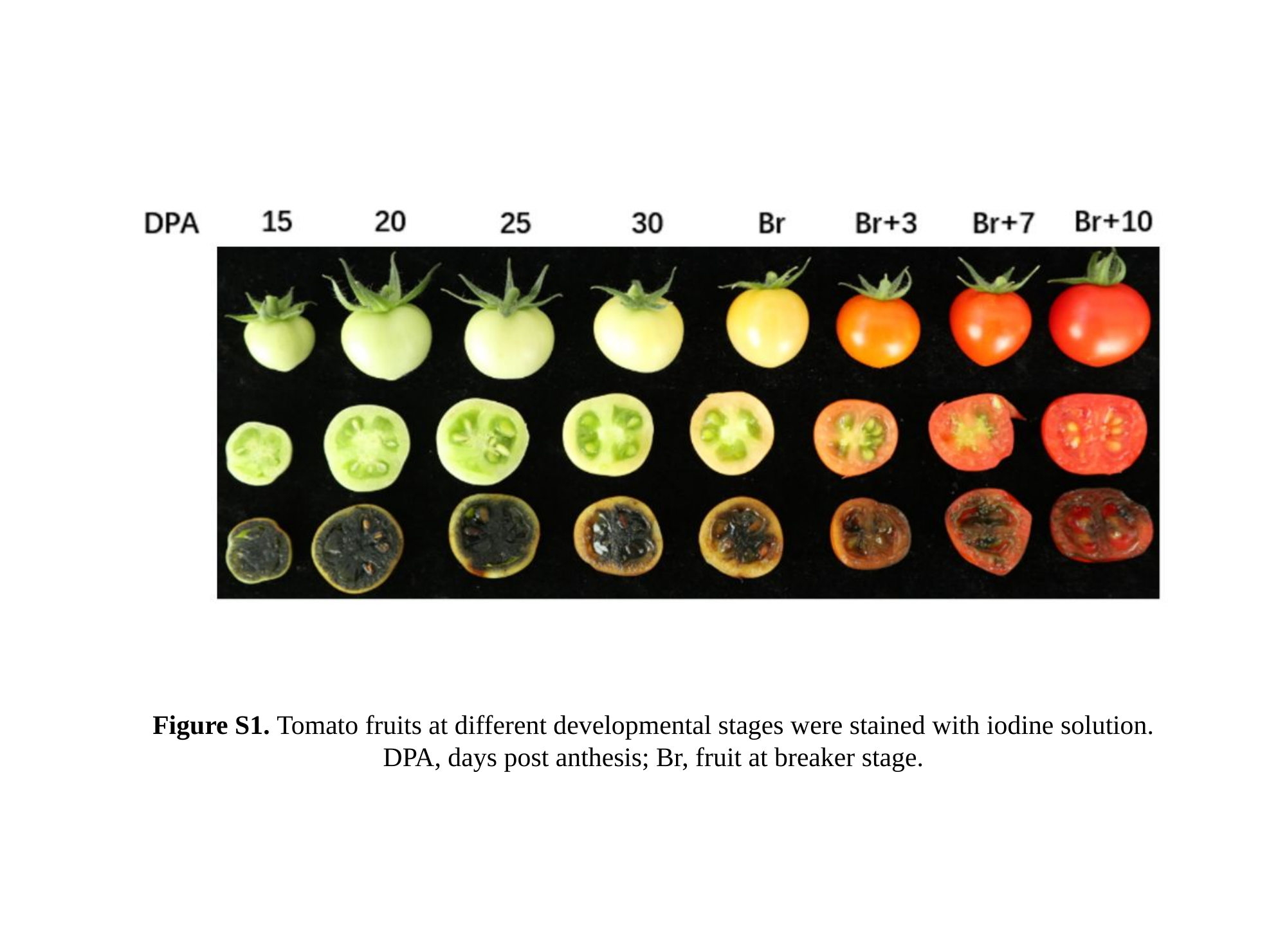

Figure S1. Tomato fruits at different developmental stages were stained with iodine solution.
DPA, days post anthesis; Br, fruit at breaker stage.

## Slide 2
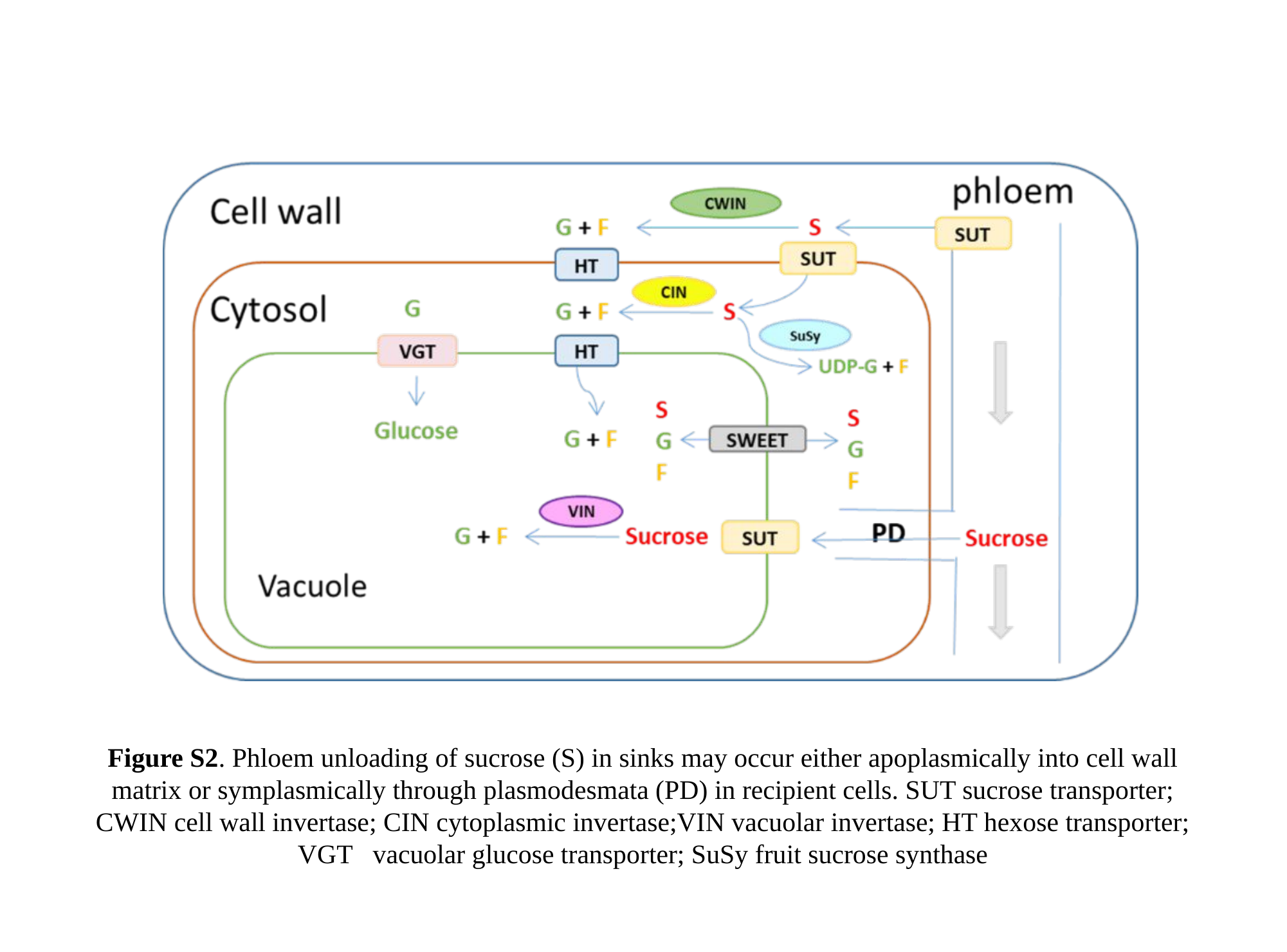

Figure S2. Phloem unloading of sucrose (S) in sinks may occur either apoplasmically into cell wall matrix or symplasmically through plasmodesmata (PD) in recipient cells. SUT sucrose transporter; CWIN cell wall invertase; CIN cytoplasmic invertase;VIN vacuolar invertase; HT hexose transporter; VGT vacuolar glucose transporter; SuSy fruit sucrose synthase

## Slide 3
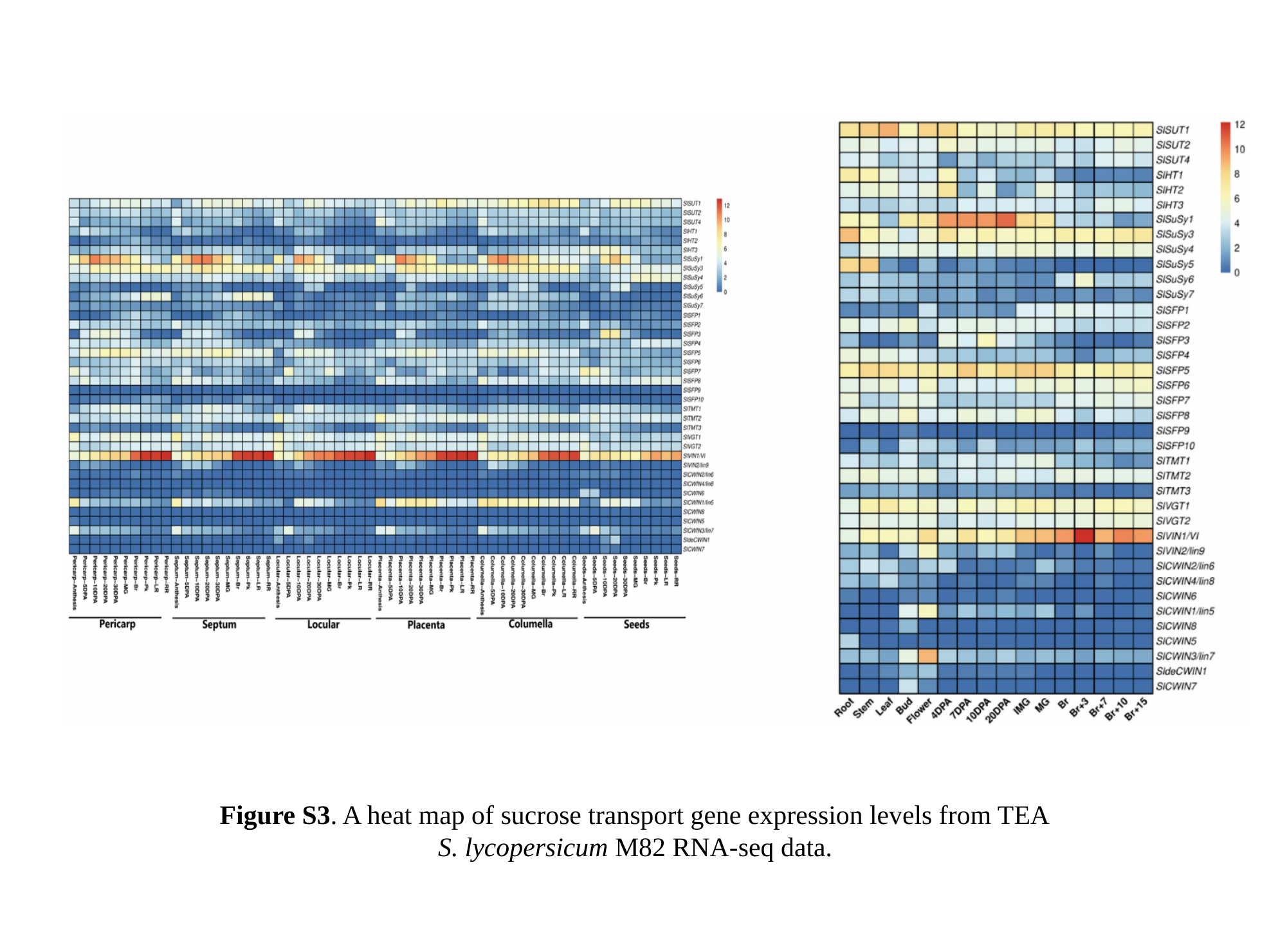

Figure S3. A heat map of sucrose transport gene expression levels from TEA
S. lycopersicum M82 RNA-seq data.

## Slide 4
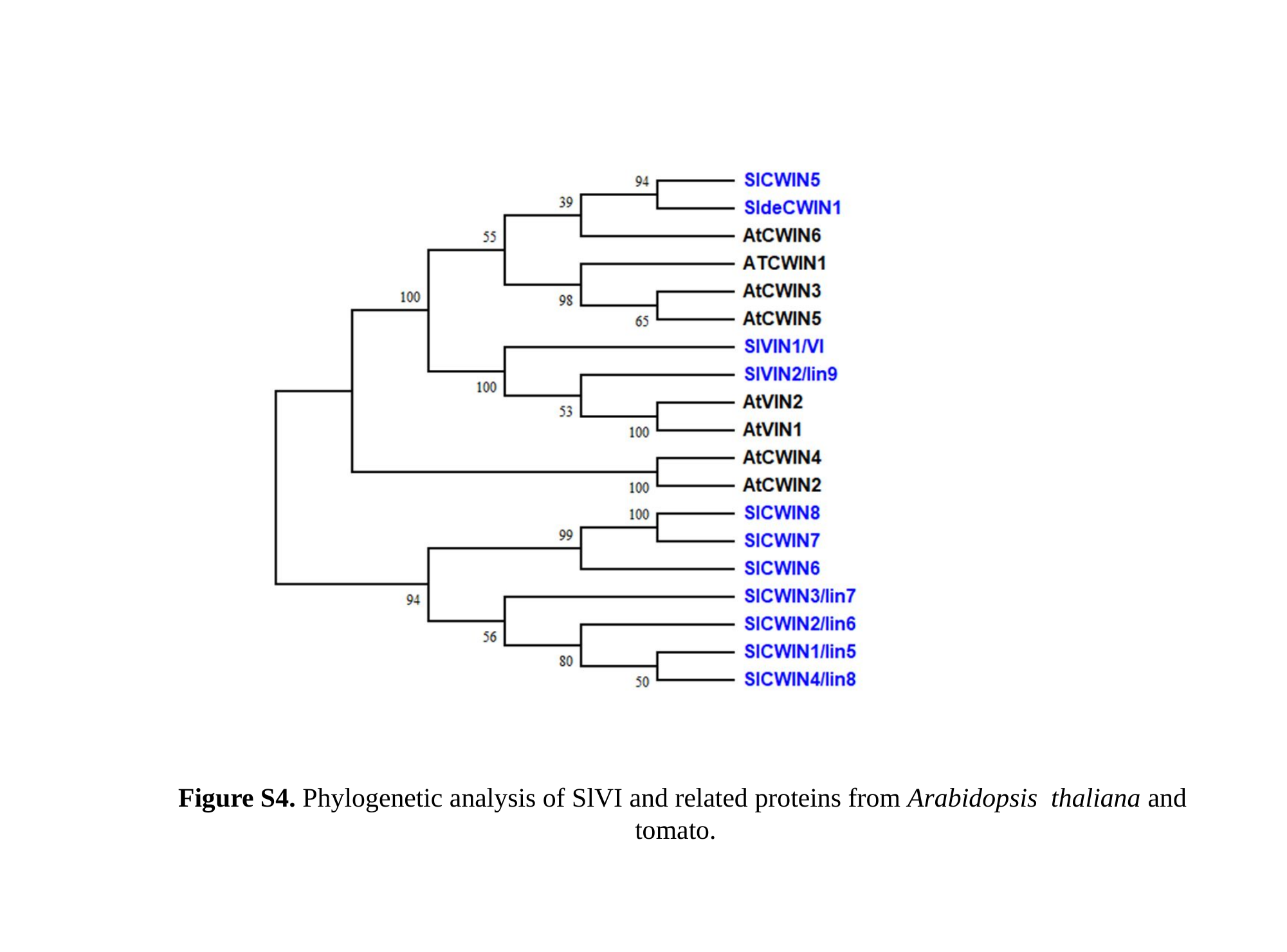

Figure S4. Phylogenetic analysis of SlVI and related proteins from Arabidopsis thaliana and tomato.

## Slide 5
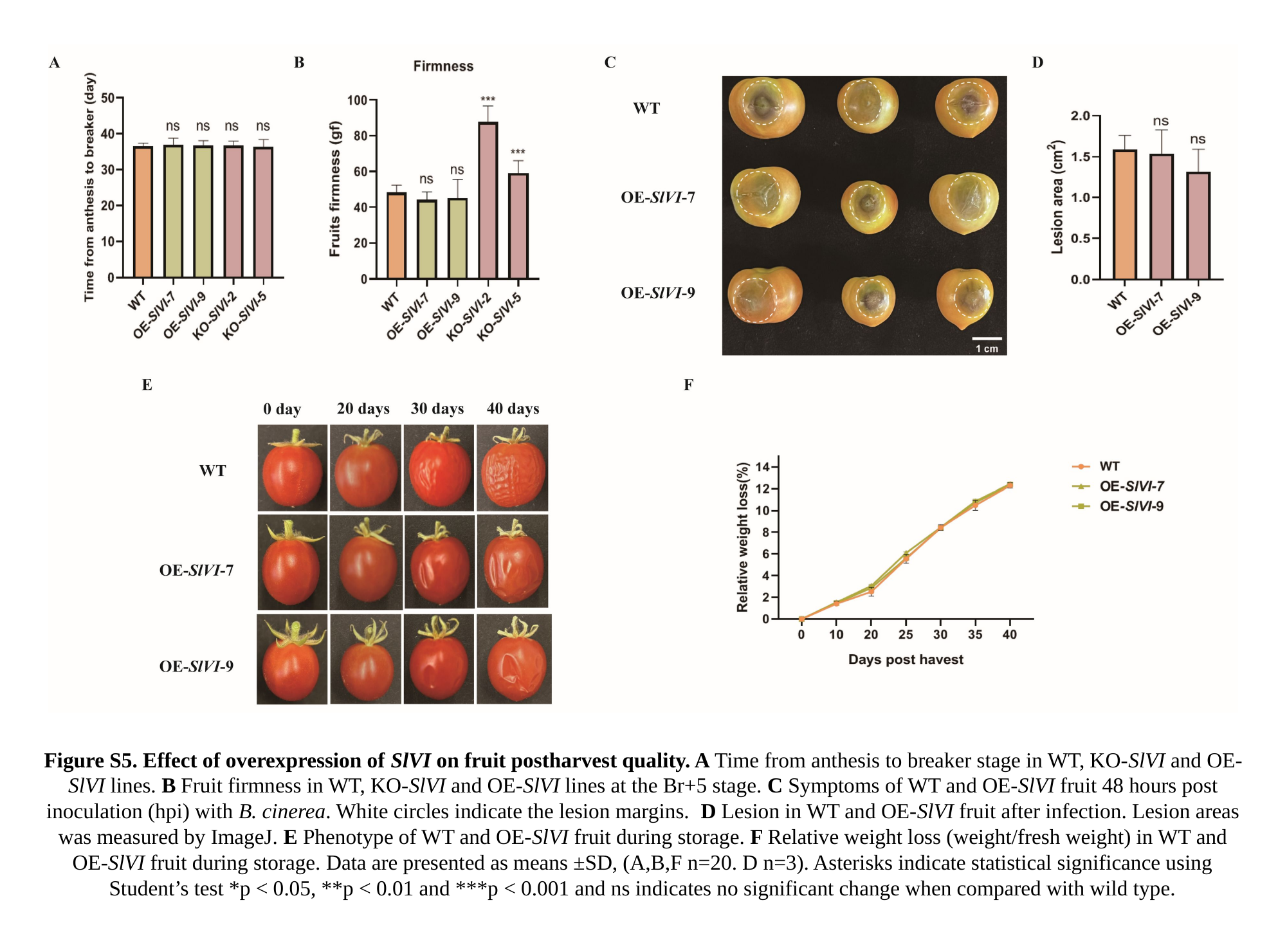

Figure S5. Effect of overexpression of SlVI on fruit postharvest quality. A Time from anthesis to breaker stage in WT, KO-SlVI and OE-SlVI lines. B Fruit firmness in WT, KO-SlVI and OE-SlVI lines at the Br+5 stage. C Symptoms of WT and OE-SlVI fruit 48 hours post inoculation (hpi) with B. cinerea. White circles indicate the lesion margins. D Lesion in WT and OE-SlVI fruit after infection. Lesion areas was measured by ImageJ. E Phenotype of WT and OE-SlVI fruit during storage. F Relative weight loss (weight/fresh weight) in WT and OE-SlVI fruit during storage. Data are presented as means ±SD, (A,B,F n=20. D n=3). Asterisks indicate statistical significance using Student’s test *p < 0.05, **p < 0.01 and ***p < 0.001 and ns indicates no significant change when compared with wild type.

## Slide 6
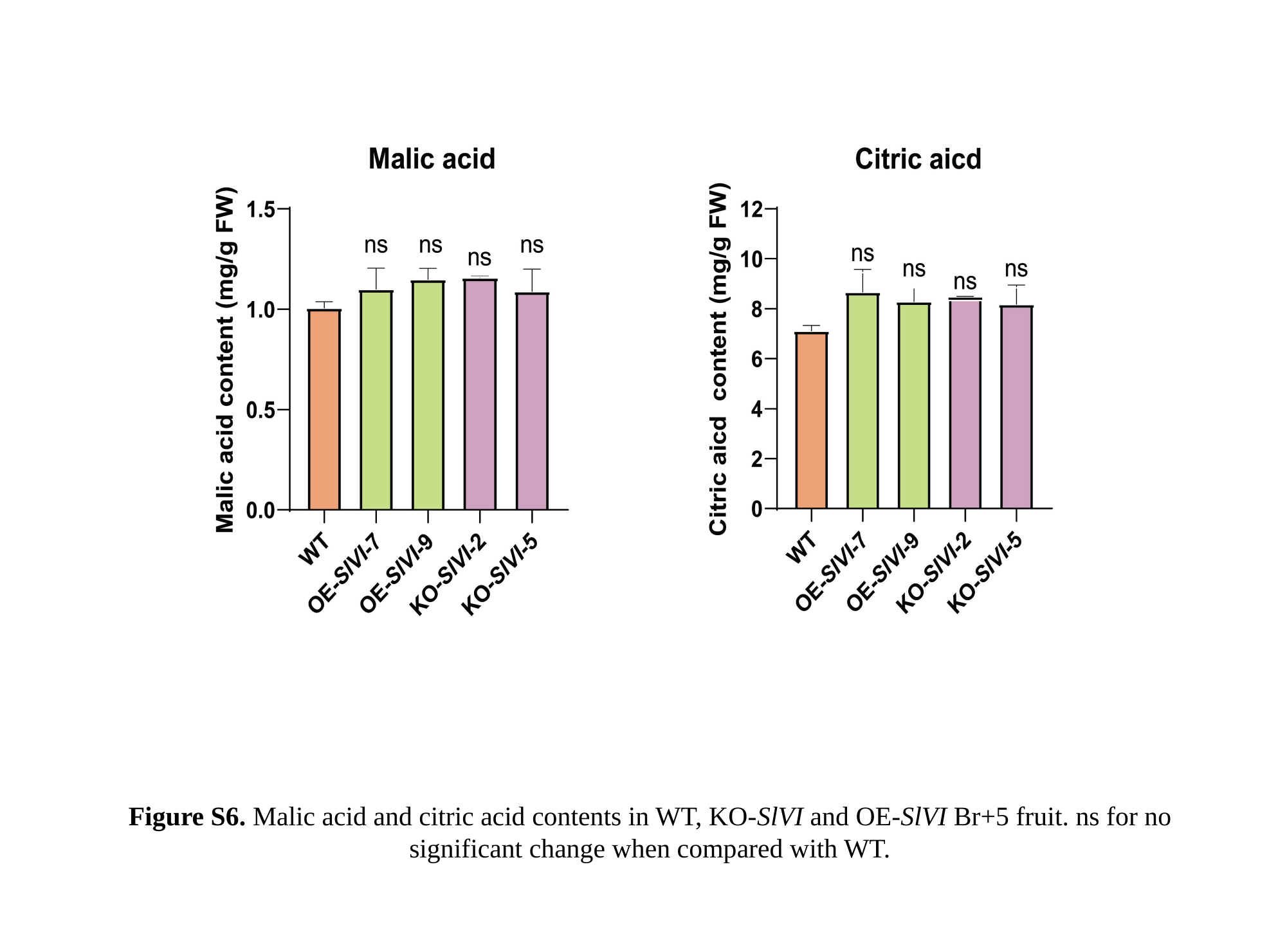

Figure S6. Malic acid and citric acid contents in WT, KO-SlVI and OE-SlVI Br+5 fruit. ns for no significant change when compared with WT.

## Slide 7
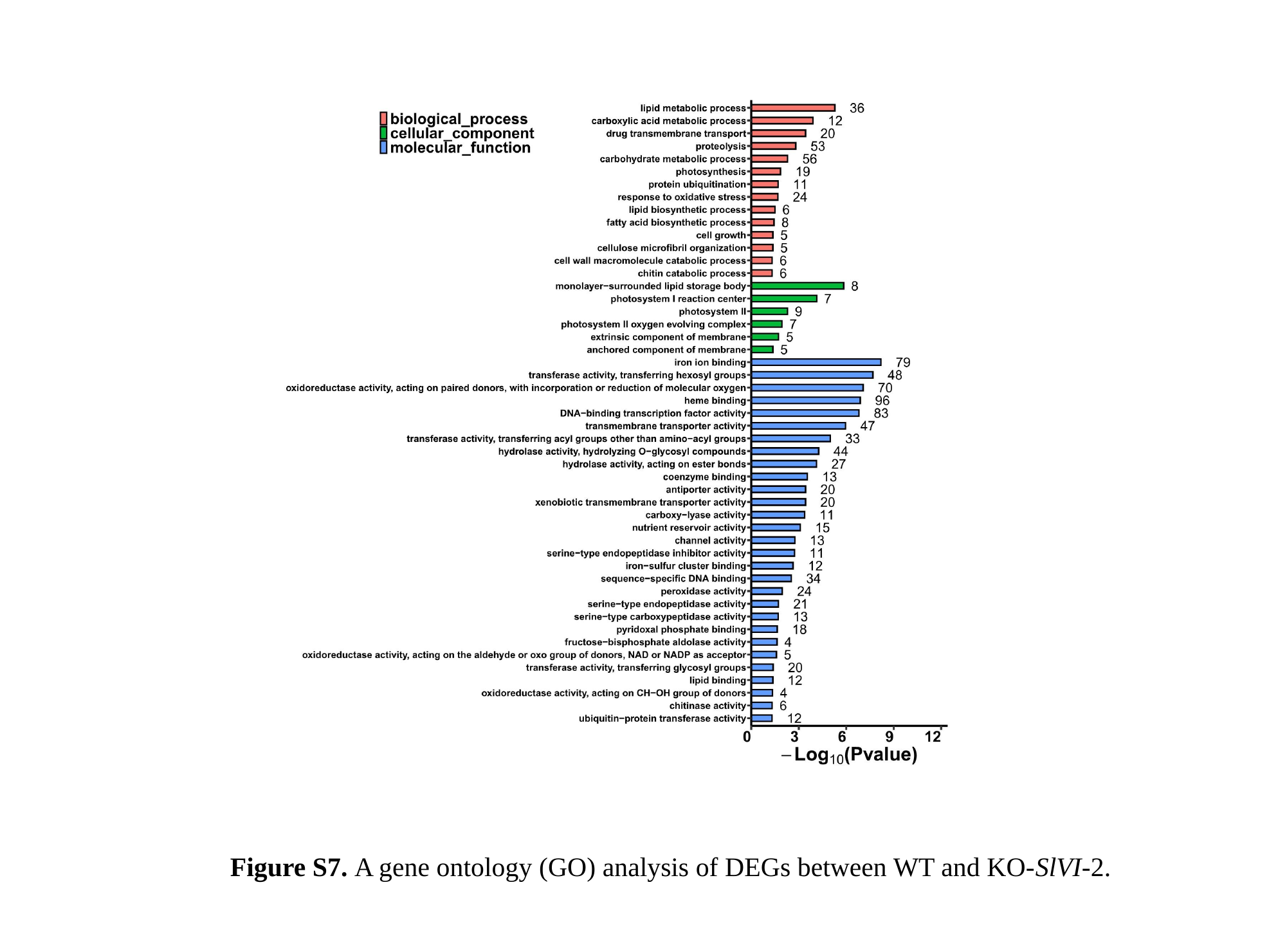

Figure S7. A gene ontology (GO) analysis of DEGs between WT and KO-SlVI-2.

## Slide 8
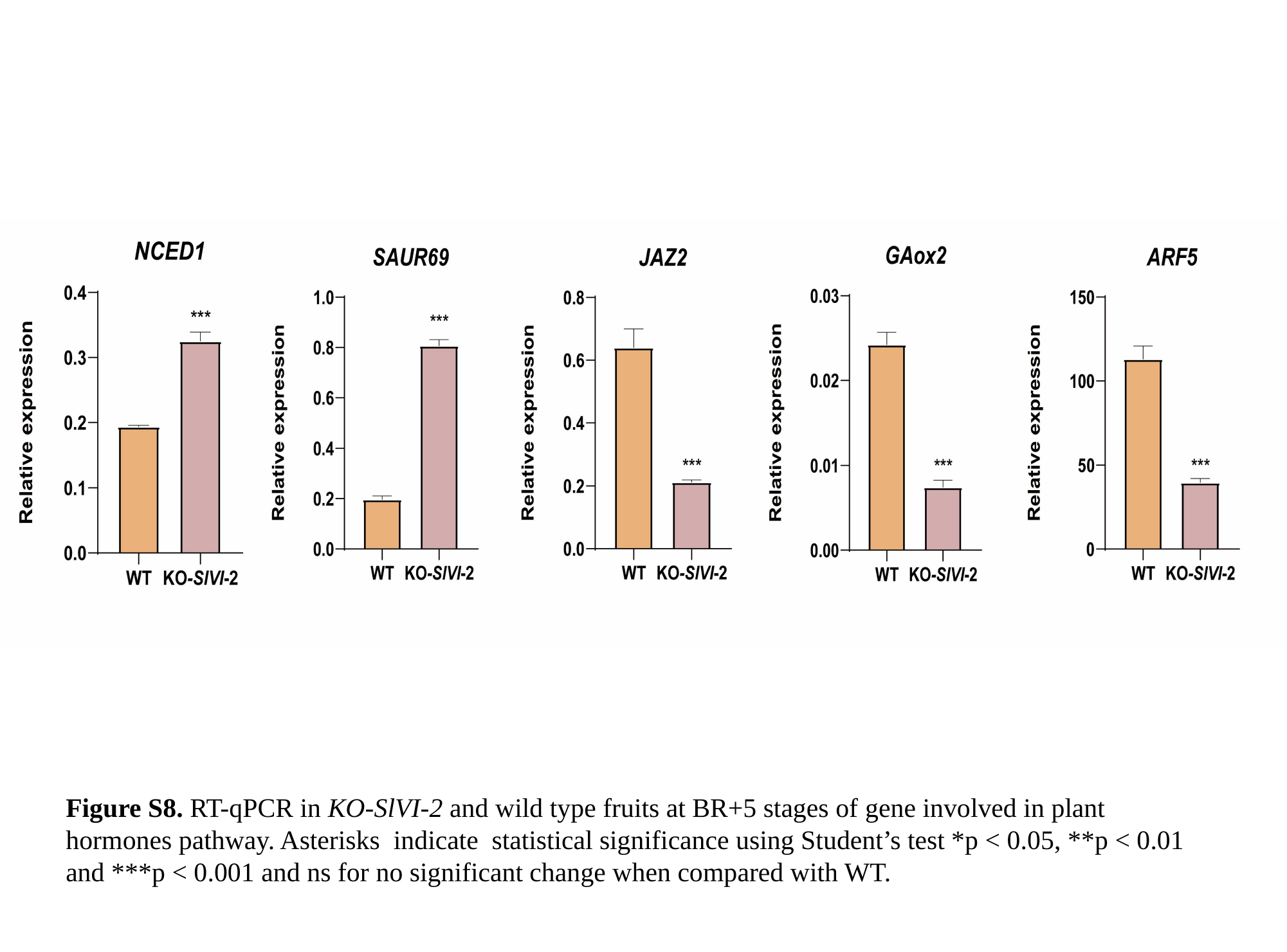

Figure S8. RT-qPCR in KO-SlVI-2 and wild type fruits at BR+5 stages of gene involved in plant hormones pathway. Asterisks indicate statistical significance using Student’s test *p < 0.05, **p < 0.01 and ***p < 0.001 and ns for no significant change when compared with WT.
